# Health Literacy, Equity, and Communication in the COVID-19 Era of Misinformation: Emergence of Health Information Professionals in Infodemic Management

**DOI:** 10.2196/35014

**Published:** 2022-04-28

**Authors:** Ramona Kyabaggu, Deneice Marshall, Patience Ebuwei, Uche Ikenyei

**Affiliations:** 1 Johnson-Shoyama Graduate School of Public Policy University of Regina Regina, SK Canada; 2 Department of Health Information Sciences Faculty of Information and Media Studies Western University London, ON Canada; 3 Division of Health Sciences Barbados Community College Saint Michael Barbados; 4 College of Health Professions, Health Information Management Coppin State University Baltimore, MD United States

**Keywords:** COVID-19, social media, infodemiology, infoveillance, equity, health literacy, digital literacy, health information management, pandemic, health information, public policy, infodemic

## Abstract

The health information management (HIM) field’s contribution to health care delivery is invaluable in a pandemic context where the need for accurate diagnoses will hasten responsive, evidence-based decision-making. The COVID-19 pandemic offers a unique opportunity to transform the practice of HIM and bring more awareness to the role that frontline workers play behind the scenes in safeguarding reliable, comprehensive, accurate, and timely health information. This transformation will support future research, utilization management, public health surveillance, and forecasting and enable key stakeholders to plan and ensure equitable health care resource allocation, especially for the most vulnerable populations. In this paper, we juxtapose critical health literacy, public policy, and HIM perspectives to understand the COVID-19 infodemic and new opportunities for HIM in infodemic management.

## Introduction

Researchers in the field of health literacy have argued that health literacy has been vastly undervalued and unrecognized in the fight against COVID-19 [1] and ought to be considered the quintessential “social vaccine” for preventing COVID-19 in populations [2]. Indeed, as an essential self-management skill and community resource for health, the effects of low population health literacy are likely to be much more pronounced under the current infodemic, in which volumes of disparate quality information are rapidly being disseminated through mediums of public communication, consumption, and information sharing.

*Health literacy* is broadly defined as the cognitive and social skills that determine individuals’ motivation and ability to gain access to, understand, and use information in ways that promote and maintain good health in a variety of settings across the life course [3]. A health literate individual can comprehend and comply with self-care instructions, plan to make changes in their lifestyle, consent to procedures, make decisions that are informed by different types of information (including quantitative health risk information), and engage in community dialogues on health and health care through lay engagement [4]. Research has shown that health literacy contributes to differences in patient knowledge, self-efficacy, self-care behavior, and health status [5-7]. It is also positively associated with vital skills that are needed for patients to function in health contexts, including the improvement of the quality and clarity of communication, patients’ involvement in clinical decision-making, patients’ willingness to express health concerns, and compliance with clinical orders [8,9]. However, despite the importance of health literacy, several countries still struggle to attain high degrees of health literacy. For instance, in Canada, an estimated 60% of adults and 88% of older adults are not health literate, and as a result, this barrier may affect their ability to make informed decisions or exert some control over their health [10]. Population health literacy also presents a concern in other high-income countries, with population health literacy levels among European countries varying widely from 71% in the Netherlands to 38% in Bulgaria [11].

*Digital literacy* can be conceived as health literacy in digital information and technology spaces [12,13]. An important point to acknowledge is that digital technology in health and social contexts presents both new risks and opportunities for equity in different information audiences. Digital health will increasingly influence social values that are based on the principles of health care systems and the experiences of those who seek health and health care. On the one hand, inequities in health are exaggerated by a widening digital divide [14]. For example, it has also been argued that digitalism and growing technocratic involvement in consumer health and health care are yet more indications of the trend toward less government involvement and more health care privatization in social democratic and liberal welfare states [15,16]. However, digital health adoption, such as the uptake of personal health records, also offers new opportunities to democratize information, improve health care navigation and access, strengthen community and social support, and reshape the patient-doctor relationship through improved communication and shared decision-making [17].

## The COVID-19 Infodemic

### Critical Health Literacy Perspective

The burden of low health literacy disproportionately affects the most socially and economically marginalized groups [18-21]. Through an intersectional lens, we can see the cumulative effects in health care through the experiences of those with low health literacy and other vulnerabilities. This can be rife with issues in navigating care; difficulties with accessing health-related information; and stigma and discrimination, which have a disempowering effect that can diminish the motivation to seek care [22]. It is not surprising then that low health literacy is associated with the greater use of emergency care, the lower utilization of preventive services, and a higher risk of poorer health outcomes, with an estimated attributable cost to health care systems of 3% to 5% of annual health care expenditures [23-26].

Nutbeam [27] first proposed a 3-tier view of health literacy—functional, interactive, and critical health literacy—whereby critical health literacy is considered the highest order of health literacy cognition and skill. de Leeuw [28] later describes critical health literacy as the “skills, capacities and knowledge required to access, understand and interact with social and political determinants of health and their social discourse.” Through critical health literacy, individuals and communities are empowered to engage in the social and political processes to jointly address the social determinants of health. Communities with high critical health literacy can strategically translate their lived health experiences into shared understandings to influence decision makers and, through collective action, address the social determinants of health that most impact them.

Although being in a more favorable socioeconomic position, including attaining higher education, is generally considered a protective factor, one of the challenges of COVID-19 misinformation and other types of health misinformation is that their effects do not just move along socioeconomic gradients. The sociocultural-driven healthism phenomenon, as defined by Crawford [29] and Greenhalgh and Wessely [30], concerns the emergence of a subculture of socioeconomically advantaged citizens who are nonetheless more likely to propagate misinformation, demand ineffective or unnecessary care, and reject high-impact health interventions under the guise of postmodern or luxury medicine. Canadians, for instance, receive more than 100 million unnecessary medical tests and treatments every year [31]. Similar trends have been found worldwide [32-34]. There is also no shortage of medical myths and misconceptions on the internet (eg, antivaccination misinformation) [35]; celebrity endorsements of harmful health products, treatments, and practices (eg, colonic hydrotherapy in general populations) [36]; organized community efforts that are in opposition to evidence-based public health measures (eg, water fluoridization) [37]; or physician reports about the pressures they receive from patients to provide treatments that have been shown to be ineffective, inefficient, or harmful (eg, inappropriate antibiotic prescribing) [38]. For individuals who engage in low-value or harmful practices, seemingly personal decisions can have broader consequences for society and the economy at large. This is especially true in the case of emerging and re-emerging infectious diseases, given the challenges of preventing or controlling them in the earliest stages. The high population-attributable risk of death due to personal exposure to COVID-19 misinformation is a reminder of such impacts [39].

The sheer volume and virality of misinformation during the current COVID-19 pandemic led the director-general of the World Health Organization (WHO), Tedros Adhanom Ghebreyesus, to declare this phenomenon an *infodemic* at the February 2020 Munich Security Conference [40]. The widespread adoption of the internet has made information more accessible. Although technology is beneficial in disseminating information rapidly, in some ways it has also played a crucial role in the dissemination of false and misleading information found on the internet, resulting in negative consequences [41]. There is also a critical health literacy aspect of this infodemic phenomenon that is often overlooked—how power and privilege manifest in the COVID-19 misinformation discourse [42]. In general, socially and economically disadvantaged groups (based on racism or ethnic identity, ableism, class, education, sexual orientation, gender identity, etc) are at a greater risk of exposure to COVID-19 [43]. Nevertheless, their voices and experiences are often sidelined. This favors those who are the least exposed to and possess more human and economic resources for bracing the impacts of the disease [44]. Making matters worse are communication inequalities. Many disadvantaged populations experience barriers to information exposure that go beyond digital access and literacy, as previously mentioned; for example, they may have fewer social ties or earn lower wages, and this requires them to work longer hours [45]. As a result, messages should be tailored based on the underlying cause of the misinformation problem, and efforts should ensue to increase people’s exposure to accurate, low-barrier, targeted health risk messaging to account for this disparity [46].

The infodemic crisis is not merely a health and digital literacy issue; it may stem from other causes, including a vulnerability to persuasive communication from broader sociocultural forces and individual psychology. When pervasive misinformation and disinformation are a problem, consideration should be given to the prime movers and beneficiaries of misinformation, who use such information to drive sociopolitical agendas and weaponize disinformation to entrench asymmetrical power, especially in times of uncertainty and threat. It can be counterproductive, when addressing the social determinants of health, to construe pervasive perceptions of attitudinal or partisan influence or identity as merely a health literacy problem. Instead, it can be acknowledged that health literacy coexists and interacts with diverse influences and, perhaps most importantly, that it can be seen as a mechanism of individual and systems change.

### Public Policy Perspective

The failure to adopt evidence-informed decision-making is not only a health spending dilemma but also, perhaps more importantly, an ethical one. According to Ciliska, Ward, Datta, and Jiwani [47], investing in treatments that do not work should be seen as an opportunity cost, which includes the direct costs diverted from doing something more effective and the indirect costs of the resultant poorer health impact. The extent to which governments communicate effectively and engage in evidence-informed decision-making plays a significant role in an individual’s acceptance of health risk messages, their perceptions of vulnerability, and the subsequent adoption and outcomes of health-protecting behaviors [48]. It is imperative that government officials and various health authorities take responsibility to ensure the reliability of COVID-19 information that is shared within public domains, especially for information in their respective jurisdictions. However, several instances can be seen in which government actors in positions of legitimate authority have demonstrated a poor recognition of misinformation, have published or disseminated inconsistent or inaccurate information, or have otherwise not adequately used evidence- and information-based decision-making processes [49].

The United Kingdom’s herd immunity strategy—an approach that relies on SARS-CoV-2 indiscriminately spreading to a critical mass in order to build up population immunity—is a particularly concerning example of evidence framing by a government [50]. When actors use scientific terminology, they can also evoke confidence and gain public trust in health policy decisions. For example, the Government of Alberta’s [51] premature and costly relaxation of COVID-19 measures, including the removal of testing and isolation, was largely established based on its premier’s, chief medical officer of health’s, and health minister’s framing and strategic use of scientific concepts and terminology. These actors declared that the province was “moving from a ‘pandemic’ to an ‘endemic’ state of COVID-19.” Indeed, most immunologists agree that an endemic state is expected at some point in the future [52]; however, Alberta’s modeling (informed by preliminary data on first-dose Delta vaccine effectiveness in the United Kingdom) did not agree with broader expert consensus [53], nor were other Canadian jurisdictions with higher population vaccine coverage rates generating similar models or making similar claims. In the end, Alberta’s endemic state measures were considered a failure. Government leaders apologized for propagating fear and anger as a fourth wave of infections overwhelmed the health care system and intensive care unit patients were transferred out of the province to receive care [54].

### Health Information Management Perspective

It is vital that during infectious disease pandemics, such as the current COVID-19 pandemic, accurate and reliable syndromic and discharge data are collected to assist with the public health response. Health information management (HIM) professionals have an enviable role in ensuring and maintaining the reliability and integrity of protected health information coming from health system encounters. According to Stanfill et al [55], “it is essential that Health Information Management (HIM) professionals ensure COVID-19 documentation, data capture, data analysis and reporting, as well as coding, are accurate and reliable to support clinical care, organizational management, public health reporting, population health management, and scientific research.” Additionally, health information managers can support contact tracing and syndromic surveillance and also assist with the mapping and forecasting of health data by applying and using various data visualization tools and techniques. Health information managers have a unique appreciation for the use of health information. HIM professionals possess the requisite skill sets for accurately coding and classifying morbidity and mortality data to validate a final diagnosis or underlying cause of death by applying the WHO rules and regulations. The health information generated has countless purposes; it supports the continuum of care and the development of targets and indicators to facilitate the planning, monitoring, and evaluation of health programs locally, regionally, and internationally. The health information produced also underwrites the development of equitable, efficient, and accessible health care systems, contributing to overall national development, which will inevitability improve public health initiatives and outcomes.

Advocating for patients and bringing attention to disparities that underlie the differential access and use of quality health information is another role in which health information managers are well positioned. Such efforts may need to start with addressing disparities in the profession, such as gender inequities and diversity within the profession, which can be seen as an indirect strategy toward building capacity for disadvantaged groups to govern and control their information to better support decision-making within communities. Beyond the profession, there has been an articulated need from racialized and ethnic minorities for more evidence on differential COVID-19 health outcomes and health system responses that is relevant to them [56,57]. The access, ownership, control, and protection of COVID-19 information have also been needs, as concerns about community privacy and risks of stigma and discrimination persist among racialized and ethnic communities [58]. As health information managers, to generate and responsibly exchange this evidence, we needed first to standardize the collection of rich, high-quality information of various types, including patient-reported experience and outcome measures and culturally appropriate, race-based, and Indigenous identity data (and this work is still in its infancy). We also needed to quickly adopt new international coding standards and work with clinicians and public health advisors serving the hardest-hit communities to improve their COVID-19 documentation practices in culturally sensitive and safe ways under the pressures and constraints of working frontline during the pandemic. The US Gravity Project, the Canadian Institute for Health Information’s Interim Standards for Race-Based and Indigenous-Identity Data Collection and Reporting, and the work of Canada Health Infoway and others on sex and gender identity terminologies could not be timelier in this regard [59-62].

The aphorism of “knowledge is power” is a useful reminder when managing an infodemic. Although HIM has been traditionally concentrated at lower levels of the Data, Information, Knowledge, Wisdom (DIKW) hierarchy, in which the veracity of knowledge is dependent, the DIKW hierarchy’s boundaries are increasingly becoming blurred. Understandings of knowledge translation may be more dynamic and data-driven than ever before due to the growing acceptance of discovery-based approaches, such as data mining and statistical modeling. In addition, advances in technology, such as artificial intelligence, are changing the way we work, allowing us to broaden our role in knowledge evaluation, management, and translation [63] and engage in more patient-facing activities. The content expertise of health information managers can serve them well as knowledge brokers who lead activities, including delivering patient-facing information triaging services; constructing user-friendly knowledge representations, such as data visualizations; and developing information interpretation tools, such as decision aids, plain language summaries, and supplementary explanatory information and metadata. In this new reality, health information managers will need to lean into their interdisciplinary underpinnings to make essential contributions in educational, informational, decision support, and behavioral informatics areas to address current and future infodemic management crises. Capacity building and skills sharing are also encouraged and are promising ways of increasing reach to individuals and communities who may not have access to the services of health information managers. Community health workers have demonstrated significant relevance in contributing to halting the spread of a pandemic and dispelling misinformation at the community level, especially in underserved communities [64]. HIM professionals can draw on the strength and reach of this cadre of health workers by building their capacity for basic documentation and information management practices. This approach ensures that information management support is available when shortages of critical human resources for health arise, as was the case at the height of the COVID-19 pandemic when the Canadian Institute for Health Information expressed the need for HIM surge capacity to support the timely capture and reporting of COVID-19 data [65].

In a recent report, the WHO Department of Infectious Hazard Preparedness outlined 5 action areas (ie, identifying evidence; translating knowledge and science, amplifying action, quantifying impact, and coordination and governance) and close to 600 specific actions to implement a comprehensive infodemic management strategy (in which strengthening health, digital, and media literacy is a significant category) [66]. Health information managers can make significant contributions to infodemic management at all levels of the DIKW hierarchy through practices such as improving the linkage and timely access to information; creating methodologies for valid and accurate data collection and analytics, especially in service of big data and artificial intelligence; and mobilizing knowledge for policy and programmatic planning. Textbox 1 provides a real-world example of an action area that health information managers are uniquely positioned to address.

**A health information manager’s role in translating knowledge and science.**

**Action area**

- Translating knowledge and science

**Specific action**

- Strengthening the interpretation and explanation of what is known, fact-checking statements, and addressing misinformation

**Case example**

- On September 1, 2020, the US White House advisor and director of the National Institute of Allergy and Infectious Diseases, Dr Anthony Fauci, appeared on the American Broadcast Company’s *Good Morning America* show to address a spurious social media claim that had gone viral via a retweet by then US President Donald Trump. The claim suggested that the Centers of Disease Control and Prevention “quietly updated” guidance on provisional COVID-19 death counts, leading the public to believe that only 6% of the over 150,000 US COVID-19 deaths reported died from SARS-CoV-2 infection, which was in fact a gross misinterpretation. In response, Fauci stated, “the point that the CDC was trying to make was that a certain percentage of [Americans who have died of COVID-19] had nothing else but just COVID. That does not mean that someone who has hypertension or diabetes who dies of COVID didn’t die of COVID-19. They did” [67].

**How can health information managers help?**

- Public demand for the near–real-time and real-time public reporting of COVID-19 data has grown; however, how mortality and morbidity statistics are reported and how they should be interpreted are not common knowledge. The above case requires an understanding of the differences between underlying and contributing causes of death. Health information managers can provide guidance and share resources [68-71] to help the general population understand COVID-19 comorbidities and clinical manifestations and how these are documented, statistically classified, and reported.

## Conclusion

Without strategies for strengthening the accuracy of judgements and individual, evidence-informed decision-making capacities, pervasive misinformation will continue to influence personal decision-making, prevent or delay public health efforts for reaching herd immunity through vaccination, and pose a threat to overall global health security, disproportionately affecting the most vulnerable and resource-limited populations.

In this paper, we present an analysis of the infodemic management crisis from critical health literacy, public policy, and information management perspectives and elucidate the role of health information managers in infodemic management responses. We argue that health information managers can draw on both technical skills and content expertise across the WHO action areas; however, as infodemiologists, they will need to reimagine how their skills can be used in different and new ways to address gaps in information quality during the era of misinformation.

Overall, combating the misinformation of the COVID-19 pandemic and any future infectious disease pandemic has to be a collaborative effort that involves all stakeholders at different decision-making levels. For example, social media outlets have a civic responsibility to verify information and to correct misinformation, and governments need to engage in evidence-informed decision-making and equip populations with the technical and cognitive tools required to interpret and use information appropriately. Health information managers are also playing a crucial role in using evidence to disseminate accurate information during this current pandemic. By using various means of improving equitable access to timely, accurate, and complete health information, health information managers are stewards of accountability, transparency, quality, and patient safety. As health information managers manage, protect, and validate the lifecycle of COVID-19 evidence (whether it be data, information, or knowledge); improve the availability of and access to relevant evidence among communities; and build individual capacity for interpreting and using evidence accurately; their work becomes further rooted in health equity. Through their work, health information managers may act as capacity builders, knowledge brokers, and agents of change in the infodemic management crisis to improve population health literacy and strengthen evidence-informed decision-making at all levels.

## Abbreviations

DIKW: Data, Information, Knowledge, Wisdom
HIM: Health Information Management
WHO: World Health Organization
