# Correction: Health Literacy, Equity, and Communication in the COVID-19 Era of Misinformation: Emergence of Health Information Professionals in Infodemic Management

**DOI:** 10.2196/35012

**Published:** 2022-05-31

**Authors:** Ramona Kyabaggu, Deneice Marshall, Patience Ebuwei, Uche Ikenyei

**Affiliations:** 1 Johnson-Shoyama Graduate School of Public Policy University of Regina Regina, SK Canada; 2 Department of Health Information Sciences Faculty of Information and Media Studies Western University London, ON Canada; 3 Division of Health Sciences Barbados Community College Saint Michael Barbados; 4 College of Health Professions, Health Information Management Coppin State University Baltimore, MD United States

In “Health Literacy, Equity, and Communication in the COVID-19 Era of Misinformation: Emergence of Health Information Professionals in Infodemic Management” (JMIR Infodemiology 2022;2(1):e35014) the authors noted one error.

In the originally published article, the degrees of author Deneice Marshall appeared as “BA, MA, Dip Education.”

In the corrected version, these degrees have been revised to “BSc, MSc, Dip Education”

The correction will appear in the online version of the paper on the JMIR Publications website on May 31, 2022 together with the publication of this correction notice. Because this was made after submission to PubMed, PubMed Central, and other full-text repositories, the corrected article has also been resubmitted to those repositories.

